# Energy Transfer in Vials Nested in a Rack System During Lyophilization

**DOI:** 10.3390/pharmaceutics12010061

**Published:** 2020-01-11

**Authors:** Sarah Daller, Wolfgang Friess, Rudolf Schroeder

**Affiliations:** 1 AbbVie Deutschland GmbH & Co. KG, Knollstraße 50, 67061 Ludwigshafen, Germany; sarah.daller@abbvie.com (S.D.); rudolf.schroeder@abbvie.com (R.S.); 2 Department of Pharmacy, Pharmaceutical Technology and Biopharmaceutics, Ludwig-Maximilians Universität München, Butenandtstrasse 5, D-81377 Muenchen, Germany

**Keywords:** lyophilization, freeze drying, rack system, heat transfer, sublimation rates, edge–vial-effect, TP, pressure, radiation, direct contact

## Abstract

Small batch sizes are a consequence of more personalized medicine and reflect a trend in the biopharmaceutical industry. Freeze drying of vials nested in a rack system is a tool used in new flexible pilot scale processing lines. Understanding of heat transfer mechanisms in the rack loaded with vials not in direct contact with each other is necessary to ensure high quality. Lyophilization in the rack vial system enables a homogeneous drying with a reduced edge-vial-effect and shielding against radiation from surrounding components, e.g., the chamber wall. Due to the separation effect of the rack, direct shelf contact contributes approx. 40% to the overall energy transfer to the product during primary drying. Hence overall the rack is a flexible, robust tool for small batch production, which ensures a controlled heat transfer resulting in a uniform product.

## 1. Introduction

Patient centered manufacturing instead of a bulk approach is trending in the biopharmaceutical industry [1]. Notably in the fields of oncology, immunology and neurology, biologics are in high demand and their contribution is still growing [2,3]. To ensure flexibility, time, and cost-effective aseptic fill/finish manufacturing at high quality, new machinery with high automation and control is being developed [4,5]. Lyophilization is often required to achieve adequate stability of the biopharmaceutical. Consequently, it is necessary to include lyophilization as part of the fill/finish process in these new flexible units which come with different vial handling compared to standard equipment using robots, disposables, ready-to-use materials, and racks.

Lyophilization is a time consuming and critical step [6]. One challenge in lyophilization is the inhomogeneous heat transfer across a shelf and related edge vial effect. During lyophilization energy can be transferred through direct contact, specifically between vial and shelf, radiation, and gas conduction. Rambhatla noted radiation from the freeze dryer walls as the main driving force for the edge vial effect [7]. It leads to higher product temperature (*T*_P_) during lyophilization and therefore higher potential for collapse. Consequently, freeze-drying processes may be run more conservative than is necessary for the vast majority of product vials. This must be considered most especially for freeze dryers with a cleaning-in-place system.

Nests with vials filled with the liquid formulation are one approach utilized in flexible automated production for transfer into the freeze dryer. This represents both challenges and opportunities which must be thoroughly understood. It is necessary to evaluate heat and mass transfer mechanisms to ensure high quality manufacturing. Especially for transfer and scale-up, understanding of energy transfer is essential to achieve adequate quality of biopharmaceutical products. The basis for determination of sublimation rates is the supposition of a steady state in heat and mass transfer as stated by Pikal et al. [8]. The contributions of gas conduction, direct contact, and radiation in a standard setting have been thoroughly summarized by Brülls and co-workers [9].

The scope of this study is to evaluate the effect of a 120-hole polyether ether ketone (PEEK) vial rack system during lyophilization on energy transfer and *T*_P_ during primary drying. The impact of the rack as well as of the lack of direct contact between the vials on modes of energy transfer were analyzed. We determined sublimation rates with water. Additionally, the drying homogeneity and the edge vial effect in the rack system were investigated. Finally, a second, smaller rack system of different material and dimensions was tested for comparison.

## 2. Materials and Methods

### 2.1. Equipment and Materials

A pilot scale freeze dryer (Hof, Lohra, Germany) equipped with four shelves with 1.0 m^2^ total surface area was used. In addition to the installed Ni/CrNi thermocouples (type K), a wireless temperature sensor system (iQ-mobile solutions, Holzkirchen, Germany) was utilized.

### 2.2. Vial Holding Systems

**Figure 1:**
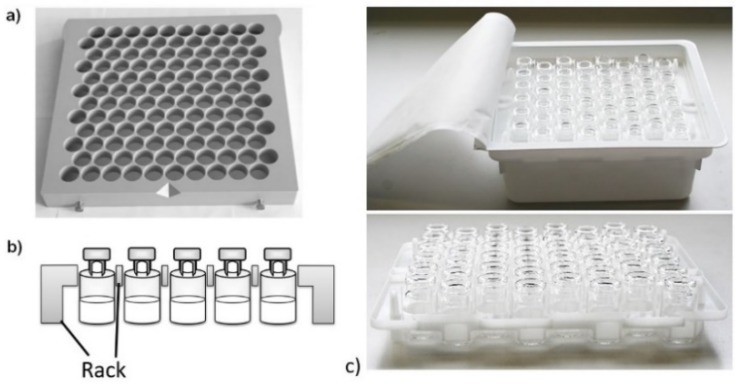
Polyether ether ketone (PEEK) rack, (**a**) top side, (**b**) cross section, (**c**) tub with nested vials from Schott, AdaptiQ.

**Figure 2:**
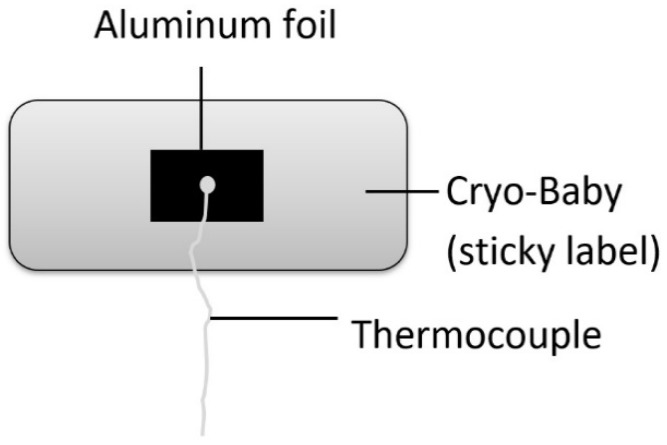
Placement of thermocouples with Cryo-Babies.

A commercial polyether ether ketone (PEEK) rack for 6R vials of 30 × 30 cm with 12 × 10 bottomless holes of 2.3 cm diameter, was used (Hof, Lohra, Germany) (Figure 1). For all experiments one fully loaded rack holding 120 6R vials was used. Additionally, a flexible 6R vial holding system of 23 × 19 cm with 8 × 6 bottomless holes, was evaluated (Schott, Mainz, Germany) (Figure 1) [10]. For temperature mapping, thermocouples were attached to the top of the rack (*n* = 8), chamber wall (*n* = 5, one at the center and four at the corners), and shelves (*n* = 18, 6 on each shelf, two at the center and four at the corners) using Cryo-Babies (sticky labels), and covered with aluminum foil (Figure 2).

### 2.3. Excipients

Either water for injection or a placebo composed of 4.6% sucrose/0.23% histidine (both from Merck, Darmstadt, Germany), pH 6.0 formulation containing 0.01% Polysorbate 80 (J.T. Baker) were used. The 6R vials (Fiolax Clear, Schott AG, Mainz, Germany) were filled with 2.5 mL. Stoppering was automatically performed in the freeze dryer at 0.5 bar nitrogen pressure with 20 mm stoppers (Dätwyler, Schattdorf, Switzerland).

### 2.4. Determination of Glass Transition Temperature Tg’ and Collapse Temperature Tc

The glass transition temperature, Tg’, of the placebo was measured using Differential Scanning Calorimetry (Netzsch, Selb, Germany) in aluminum crucibles during heating from −75 °C to 20 °C at 10 K/min (*n* = 3). The collapse temperature, Tc, was measured by freeze drying microscopy (Biopharma Systems, Winchester, UK), cooling the sample to −40 °C at 20 K/min, applying 0.001 mbar vacuum, and heating to 20 °C at 0.25 K/min (*n* = 5).

### 2.5. Freeze Drying Procedure

Samples were frozen to −45 °C and primary drying was performed at −25 °C, followed by secondary drying at 25 °C both at 0.066 mbar (Table 1).

All temperature ramps were performed at 1 K/min. Corner vials were defined as vials with fewer neighbors than center vials, which were arranged in a hexagonal neighbor packaging. *T*_P_ was measured with thermocouples or wireless sensors placed at the bottom center of the vials according to literature [11].

### 2.6. Determination of Sublimation Rates

Sublimation rates (*n* = 1 both for rack and separated vials) were determined with water by weighing all 120 vials before and after freeze drying. Samples were frozen to −40 °C. Sublimation was performed at 5 °C shelf temperature (*T*_shelf_) for 7 h at 0.066, 0.133, 0.200, and 0.267 mbar. Vials were also weighed after freezing and evacuation only. For sublimation rate (*dm*/*dt*) determination, the mass loss per vial (*m*_t_) was corrected for the mass loss per vial after freezing and evacuation only (*m*_e_) with *t* = 7 h according to Equation (1).

(1) $$\frac{dm}{dt}=\frac{\left(m_{t}-m_{E}\right)}{t}$$

For temperature measurements one wireless sensor for center temperature and one for corner temperature were placed at a corner and a center positioned vial.

### 2.7. Modes of Energy Transfer and Impact of the Rack

To investigate the impact of the rack on heat transfer, *T*_P_ was measured in separated vials. For this purpose, vials were placed in the rack and afterwards the rack was removed while the vials remained in the same arrangement. To analyze the contribution of direct contact between shelf and vial, a 0.5 cm Styrofoam (extruded polystyrene) plate was paced under the vials standing in a rack and sublimation rates were determined. The heat transfer coefficient, *K*_v_, was determined from sublimation rate, heat of sublimation of ice (Δ*H*_s_), the vial outer cross-sectional area (*A*_v_), the shelf surface temperature (*T*_s_), and the temperature at the center bottom of the vial (*T*_b_) [8,12] according to Equation (2).

(2) $$K_{v}=\frac{\frac{dt}{dm}\cdot \Delta H_{s}}{A_{v}\cdot \left(T_{s}-T_{b}\right)}$$

## 3. Results and Discussion

### 3.1. Characterization of the Solution 

The glass transition temperature of the placebo was −30.5 °C with an onset at −32.4 °C. The collapse temperature was similar to −33.0 °C. To stay well below the critical product temperature, the freeze drying cycle mentioned in the method section was employed. Edge vials, which are known as the hot spots in a batch [12] showed a *T*_P_ of −35 °C and no collapse during primary drying.

### 3.2. Behavior of the Rack during Rreeze Drying

**Figure 3:**
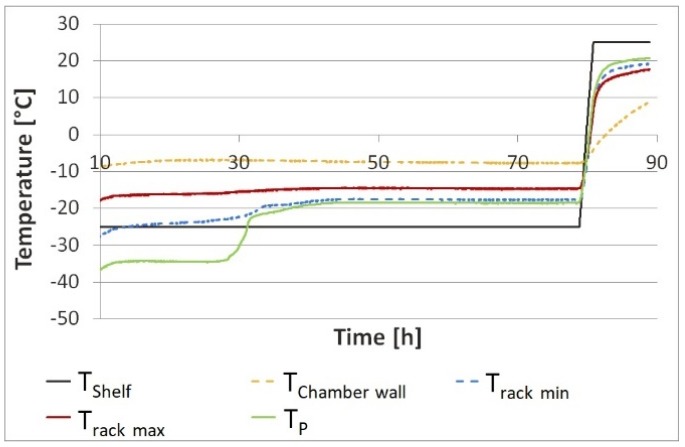
Temperature mapping: cold and hot spots of the rack in comparison to *T*_P_ and *T*_chamber wall_.

Temperature mapping showed higher temperature at the corners of the top side of the rack and lower temperatures at the bottom side of the grid differing in temperature by approx. 10 °C (Figure 3). Temperatures of the rack, product, and chamber wall are summarized in Table 2.

During primary drying the top of the rack was 10 °C warmer than the shelves and the bottom side of the grid was −23 °C. The rack, especially the outside of the rack is impacted by the radiation coming from the warmer chamber wall. Due to the low heat transfer coefficient of PEEK of 0.25 W·m^−1^·K^−1^ [13], the rack adapts to *T*_shelf_ slowly. During primary drying, energy transfer from the rack to the vial via radiation and gas conduction is to be expected. In the primary drying phase, the chamber wall was approx. 10 °C warmer than the rack which itself is warmer than the product by 10–20 °C. At the same time the massive rack reduced radiation from the wall directly onto the product, potentially reducing the edge vial effect.

### 3.3. Modes of Energy Transfer in Separated Vials

**Figure 4:**
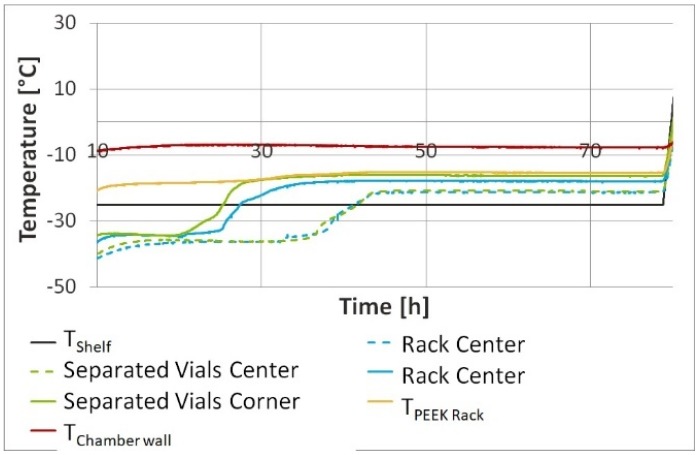
*T*_P_ of separated vials compared to *T*_P_ of vials in the rack. Definition of separated vials: vials positioned on a shelf with the aid of the rack but with the rack removed after positioning.

Separated vials, meaning vials positioned on a shelf with the aid of the rack but with the rack removed after positioning, showed an earlier beginning of the endpoint of sublimation in primary drying (Figure 4). Temperatures of the rack, the chamber wall and product in both the rack and separated vials are summarized in Table 3.

**Figure 5:**
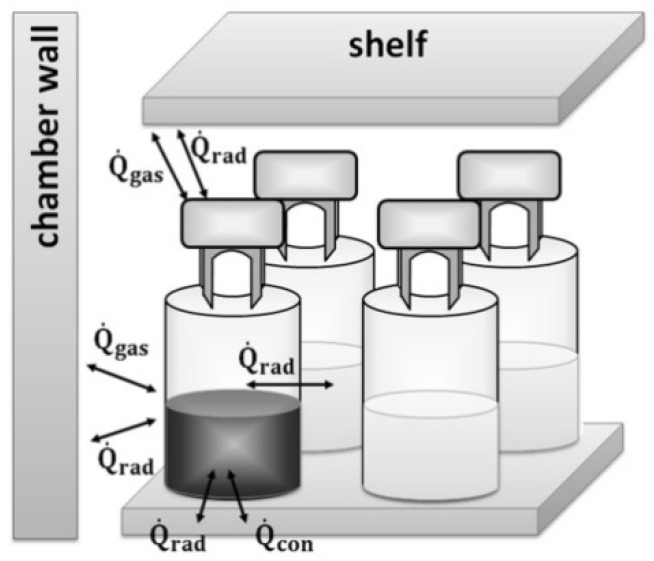
Energy transfer of a separated corner vial.

In both settings the center vials had a similar behavior during freeze drying and reached an endpoint after 45 h. For corner vials in the rack the steady state of primary drying ended after 25 h as compared to 20 h for separated vials. In separated vials the energy transfer in primary drying mainly involves direct contact with the shelf, radiation coming from the chamber wall, and gas convection (Figure 5).

Lacking a radiation shield, separated corner vials dried at a 3 °C higher temperature during primary drying as compared to vials standing in a rack. The difference in drying time between corner and center vials in the rack was 27% as compared to 40% in the case of separated vials without a rack. Thus, the rack enables a homogeneous *T*_P_ and primary drying of corner and center vials.

### 3.4. Energy and Mass Transfer in a Rack System

**Figure 6:**
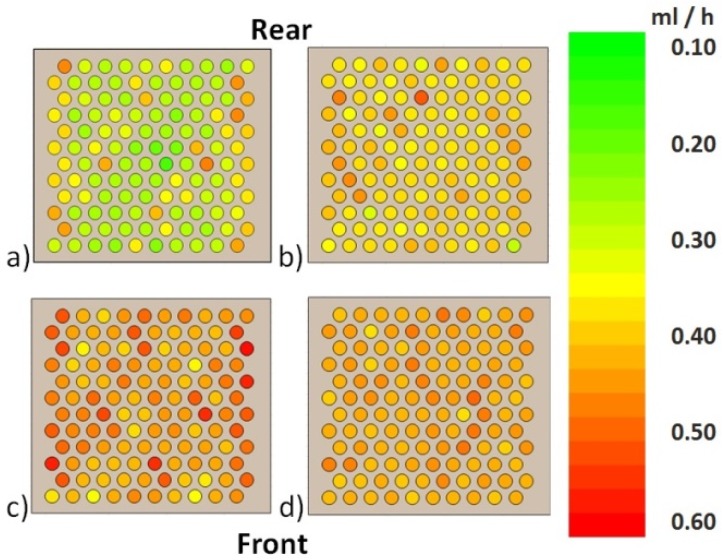
Mapping of sublimation rates in the rack at (**a**) 0.066 mbar, (**b**) 0.133 mbar, (**c**) 0.200 mbar, and (**d**) 0.267 mbar.

Sublimation rate mapping of vials filled with water in the rack at 0.066 mbar chamber pressure and *T*_shelf_ of −25 °C showed that the mass transfer in corner vials is 24% higher than in center vials (Figure 6).

**Figure 7:**
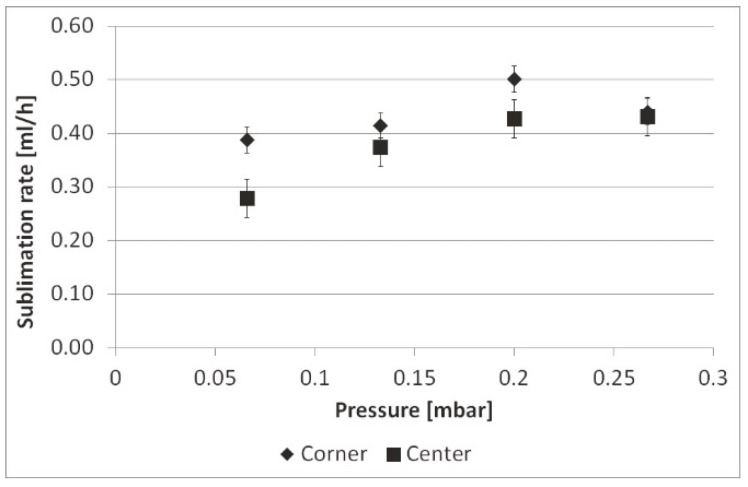
Mean sublimation rates in the rack at 0.066 mbar, 0.133 mbar, 0.200 mbar and, 0.267 mbar.

Corner vials left and right showed higher sublimation rates and 3–4 °C higher *T*_P_ compared to corner vials at the front and rear sides. The mean sublimation rates at 0.066, 0.133, 0.200, and 0.267 mbar (*n* = 1) are shown in Figure 7.

**Figure 8:**
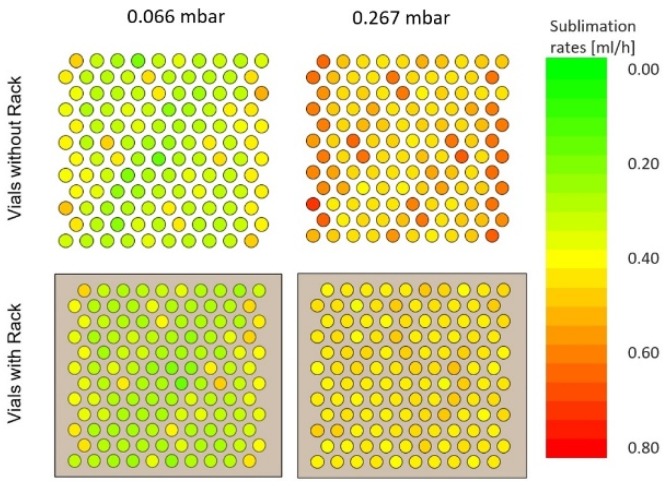
Sublimation rate mapping for vials with and without a rack at 0.066 mbar and 0.267 mbar.

As illustrated by Pisano et al., sublimation rates increase with increasing pressure [14]. At 0.066 mbar the sublimation rates between corner and center differed by 28%. These differences diminished with a higher pressure of 0.267 mbar to 2.3%. Due to a lack of direct contact between the vials, gas molecules are able to reach edge and center vials in the same manner. With increasing pressure, gas conduction becomes a stronger contributor to heat transfer [9], resulting in reduced edge effect. Separated vials in the same arrangement without a rack showed higher sublimation rates as they were not shielded from radiation (Figure 8).

The difference between corner and center vials was 31% at 0.066 mbar, which decreased to 22% at 0.267 mbar.

**Figure 9:**
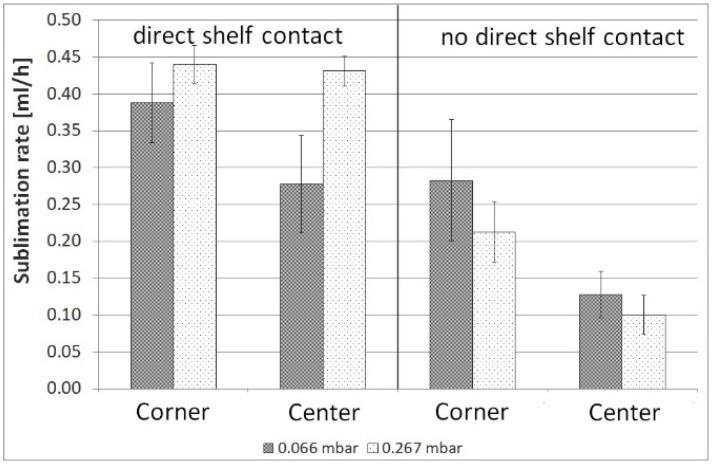
Mean sublimation rates in center and edge vials, either with direct contact to the shelf or without, at 0.066 mbar, error bars represent standard deviation (*n* = 10).

Ganguly et al. found a 17% contribution from direct contact between vial and shelf to the total heat transfer at low pressures which decreased to 10% at high pressures [15]. An additional experiment with a Styrofoam plate placed under the vials was performed. Due to the low thermal conductivity of 0.029 W·m^−1^·K^−1^ [16], the Styrofoam plate was assumed to minimize energy transfer via direct contact to a minimum. The sublimation rate of 0.066 mbar decreased by 28% for corner vials and 54% for center vials (Figure 9).

Therefore, the contribution of direct contact to the total heat transfer in the rack can be assumed to be 42% on average. At 0.267 mbar, sublimation rates decreased by 52% in corner vials and by 77% in center vials when direct contact between vial and shelf was prevented by a Styrofoam plate. Contribution of direct contact to total heat transfer is higher at higher pressures. In the rack, the heat transfer via direct contact is reduced to a vial-to-shelf contact. For center vials, the impact of radiation from the chamber wall becomes negligible due to surrounding vials.

### 3.5. Comparison of the Rack System to Another Nested Vial System

The utilization of flexible small-scale manufacturing lines makes vial holding systems necessary. We consequently evaluated another flexible holding system of different geometry and made of polyoxymethylene (POM) instead of PEEK. The smaller POM nest system had no contact with the shelf and no band surrounding the vials. Including 24 corner and 24 center vials, the ratio of corner to center is higher compared to the rack, which includes 40 corner and 80 center vials.

**Figure 10:**
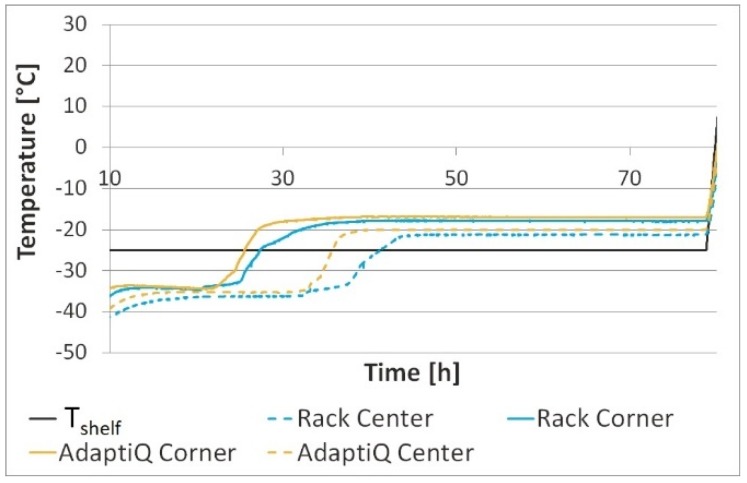
*T*_P_ of vials in a rack and AdaptiQ nest.

Compared to corner and center vials standing in the PEEK rack system, for which primary drying ended after 24 h and 36 h respectively, the steady state of primary drying ended 10% earlier, after 21 h for corner and 31 h for center vials, in the flexible POM nest (Figure 10).

There is less shielding from radiation provided by the POM nest compared to the rack. In AdaptiQ, radiation coming from the chamber wall is able to impact vials at the corners. Therefore, AdaptiQ corner vials behave similarly to separated corner vials without a rack.

## 4. Conclusions

Heat transfer for sublimation in vials nested in a rack system is dominated by direct contact between vial and shelf and radiation coming from the rack itself. Heat transfer through direct contact is limited to contact between vial and shelf. Contribution of direct contact is higher and radiation effect from the chamber wall is less than in the standard configuration. This allows a reduction in the difference of *T*_P_ between corner and center vials from 39% to 27%. Separated corner vials without a rack showed a 6 h shorter primary drying time as they lacked the radiation shielding provided by the rack. With increasing pressure, the difference in sublimation rates between corner and center vials in the rack decreased due to a higher contribution of gas conduction, leading to a reduced edge-vial-effect. Compared to another smaller, more flexible, nested vial system without shelf contact, the primary drying time is reduced by 10%. Finally freeze drying of vials nested in the rack system is an important tool in flexible manufacturing units which require a good understanding of heat transfer. They can provide a controlled heat transfer with reduced edge vial effect. Future research will investigate a 1:1 comparison of rack with bulk setting and focus on transfer of freeze-drying cycles within and between different vial arrangements.

## Figures and Tables

**Table 1:** Freeze drying cycle for temperature measurement experiments.

Step No.	Time (hh:mm)	Temperature (°C)		Vacuum (mbar)
		Shelves	Condensor	
1 Loading	00:01	20.0	n/a	1000.0
2 Freezing	00:20	0.0	n/a	1000.0
3 Freezing	02:10	0.0	n/a	1000.0
4 Freezing	01:20	−45.0	n/a	1000.0
5 Freezing	3:00	−45.0	n/a	1000.0
41 Evacuation	01:00	−45.0	−85.0	0.066
42 Primary Drying	70:00	−25.0	−85.0	0.066
43 Primary Drying	02:00	−25.0	−85.0	0.066
92 Secondary Drying	00:15	25.0	−85.0	0.036
93 Secondary Drying	08:00	25.0	−85.0	0.036
94 Secondary Drying	00:20	5.0	−85.0	0.036
95 Storage	00:01	5.0	−85.0	0.036

**Table 2:** Temperatures of rack, product, and chamber wall during primary drying.

Position	Temperature (°C)
Top side of the rack	−15
Bottom side of the rack	−23
Shelf	−25
Chamber wall	−8
Product	−35

**Table 3:** Measured *T*_P_ in the rack compared to separated vials.

Position	Temperature (°C)		End of Steady State Phase (h)	End of Primary Drying (h)	Difference between Corner and Center at the End of Primary Drying (%)
	During Steady State Phase	At the End of Primary Drying			
Rack	−20	−15	n/a	n/a	n/a
Shelf	−25	−25	n/a	n/a	n/a
Chamber wall	−8	−8	n/a	n/a	n/a
Separated vials corner	−33	−15	20	27	40
Separated vials center	−35	−21	33	45	
Rack corner	−33	−18	25	33	27
Rack center	−35	−21	33	45	
